# Genome Sequence of Type Strain *Lysinibacillus macroides* DSM 54^T^

**DOI:** 10.1128/genomeA.01271-15

**Published:** 2015-11-05

**Authors:** Guo-hong Liu, Bo Liu, Jie-ping Wang, Jian-Mei Che, Qian-Qian Chen, Zheng Chen, Ci-bin Ge

**Affiliations:** Agricultural Bio-Resources Research Institute, Fujian Academy of Agricultural Sciences, Fuzhou, Fujian, China

## Abstract

*Lysinibacillus macroides* DSM 54^T^ is a Gram-positive, spore-forming bacterium. Here, we report the 4,866,035-bp genome sequence of *Lysinibacillus macroides* DSM 54^T^, which will accelerate the application of degrading xylan and provide useful information for genomic taxonomy and phylogenomics of *Bacillus*-like bacteria.

## GENOME ANNOUNCEMENT

Previously, there were two strains named *B. macroides*, namely, ATCC 12905^T^ (= DSM 54^T^ = LMG 18474^T^), on which the original description was based, and NCIMB 8796 (= NCDO 1661 = LMG 18508). The latter was considered to belong to *Bacillus simplex* by Heyrman et al. (1). *B. macroides* DSM 54^T^ conforms to the original description of this species and was concluded to be the true *B. macroides*. In 2007, Ahmed et al. (2) transferred the closest relatives of *B. macroides* to *Lysinibacillus* as *L. sphaericus* and *L. fusiformis*, and also described the novel species *L. boronitolerans* (1). *B. macroides* was proposed to be one species of the genus *Lysinibacillus* by Coorevits et al. (3) through DNA-DNA relatedness and the peptide type of the cell wall, and named *Lysinibacillus macroides* DSM 54^T^. Here, we present a summary classification and a set of features for *Lysinibacillus macroides* DSM 54^T^ together with the description of the genomic sequencing and annotation.

The genome sequencing of *L. macroides* DSM 54^T^ was performed via the Illumina Hiseq 2500 system. Two DNA libraries with insert sizes of 500 and 5,000 bp were constructed and sequenced using the 2 × 150 bp paired-end sequencing strategy. The genome coverage was approximately 150-fold coverage. The reads were assembled via the SOAPdenovo software version 1.05 (4), using a key parameter K setting at 31. Through the data assembly, 15 scaffolds with total length 4,866,035 bp were obtained, and the scaffold N_50_ was 1,112,050 bp. The average length of the scaffolds was 324402 bp, and the longest and shortest scaffolds were 1,532,948 bp and 670 bp, respectively.

The annotation of the genome was performed using the NCBI Prokaryotic Genomes Automatic Annotation Pipeline (PGAAP) (http://www.ncbi.nlm.nih.gov/genome/annotation_prok/) utilizing GeneMark, Glimmer, and tRNAscan-SE tools (5). A total of 4,717 genes were predicted, including 4,371 coding sequences (CDS), 251 pseudo genes, 86 tRNAs, 9 rRNA genes, and 66 frameshifted genes. The average DNA G+C content was 37.88%, with a slight difference to the value 38.2 mol% acquired by HPLC determination (3).

### Nucleotide sequence accession numbers.

This whole-genome shotgun project has been deposited at DDBJ/EMBL/GenBank under the accession no. LGCI00000000. The version described in this paper is version LGCI00000000.1.
